# A Hematoma Block in the Wrist for a Displaced Distal Radius Fracture Induces Systemic Neurological Symptoms: A Case Report

**DOI:** 10.7759/cureus.42921

**Published:** 2023-08-03

**Authors:** Christopher S Burch, Christopher G Stevens

**Affiliations:** 1 Orthopaedic Surgery, Tucson Orthopaedic Institute, Tucson, USA; 2 Hand/Upper Extremity Division, Tucson Orthopaedic Institute, Tucson, USA

**Keywords:** closed fracture reduction, neurologic complications, fracture of distal radius, lidocaine toxicity, hematoma block

## Abstract

Hematoma blocks are considered a relatively simple, safe, and effective technique in the acute setting to provide sufficient anesthesia and allow for closed reduction of displaced distal radius fractures. Complications associated with the utilization of local anesthetics in this setting are rare. We present the case of a patient who had a hematoma block in the wrist and developed short-term systemic neurologic complications likely secondary to systemic absorption of 20 mL of 1% lidocaine without epinephrine via the cancellous bone channels.

## Introduction

Distal radius fractures (DRFs) are one of the most well-recognized and common bony injuries treated in the acute care setting across the United States, accounting for 17.5% of all fractures [1]. The etiology, pathophysiology, and natural history of non-operative and operative treatment of DRFs are well understood [1-3]. In general, DRFs tend to have a higher incidence in older populations (60 and older), are more likely due to low-energy trauma, and are more commonly seen in females, corresponding with increased susceptibility to osteoporosis [1]. In the acute setting, the standard of care is to attempt a closed reduction if significant displacement exists. By attempting a closed reduction, the provider can take the tension off the soft tissues, improve the radiographic alignment, and give the patient a chance to do well with non-operative care.

Conscious sedation using ketamine, manual manipulation under general anesthesia, bier block, and hematoma blocks are standard anesthetic techniques used to reduce distal radius fractures [3,4]. The goal is to provide sufficient anesthesia so the patient can relax, allowing for an attempted closed reduction while minimizing patient discomfort. The most common technique is to perform a hematoma block into the fracture site. This has the benefit of minimizing patient sedation and adverse reactions and not requiring the number of staff that conscious sedation techniques often necessitate (such as respiratory therapists) [4]. Hematoma blocks are commonly performed in the acute setting using a local anesthetic such as lidocaine or bupivacaine, where the medication is placed directly into the fracture hematoma. This allows for anesthesia to be imparted to the fractured area while preserving the motor and sensory functions of the hand. After the hematoma block has been set, a closed reduction can often be performed with minimal discomfort to the patient.

To our knowledge, few authors have reported complications associated with hematoma blocks in the wrist. This case report focuses on a patient with a dorsally displaced and translated distal radius fracture in exquisite pain who had a hematoma block performed in an outpatient clinical setting and experienced short-term systemic neurological effects.

## Case presentation

**Figure 1:**
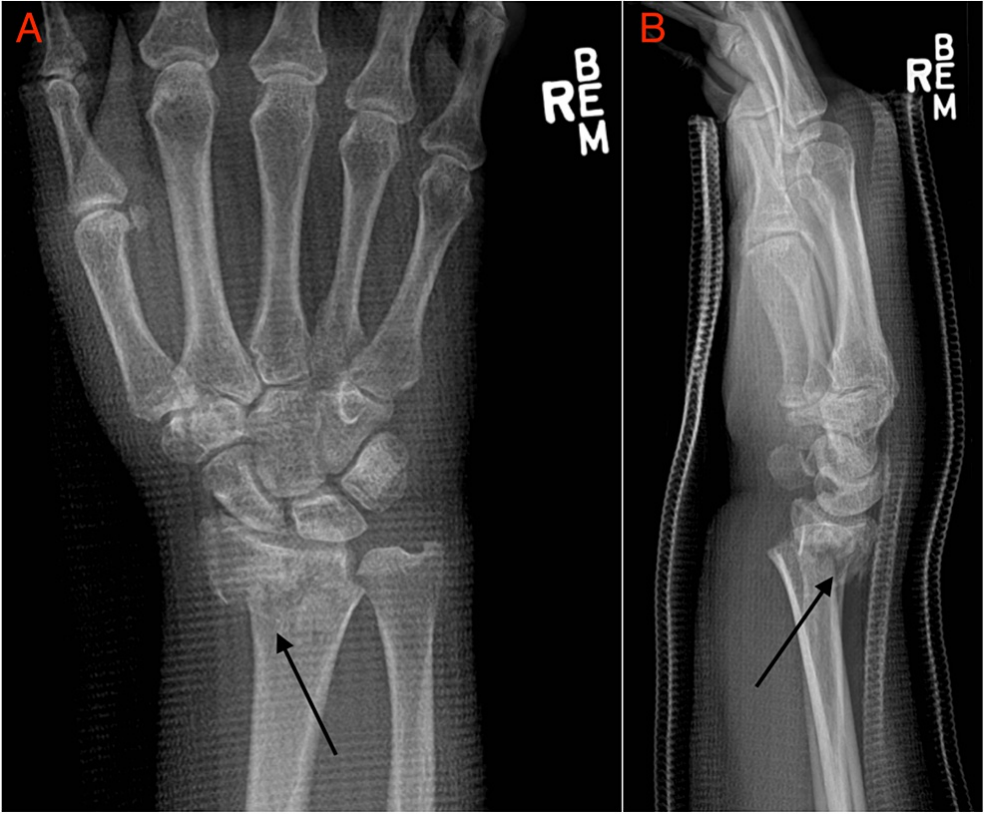
Posteroanterior (A) and lateral (B) radiographs of the right wrist were performed in the emergency department prior to closed reduction. There is an extra-articular distal radius fracture with dorsal and radial translation of the distal fragment and approximately 20 degrees of dorsal angulation.

A 72-year-old right-handed female weighing 81.65 kg with no prior personal or family history of neurological events was evaluated in the outpatient clinic for right wrist pain following a mechanical fall. The patient had been seen in the emergency department (ED) a few days before visiting the outpatient clinic. During her visit to the ED, she reported significant pain and a closed reduction was performed, resulting in considerable pain alleviation and improvement in the radiographic parameters of her fracture. However, over the days following this, the patient’s pain increased, and radiographs performed in the outpatient clinic confirmed loss of the reduction with significant dorsal angulation and translation of the fracture (Figure 1).

The dorsal angulation was measured at that time to be 20° with significant radial translation. Given the unstable nature of the fracture and the loss of reduction, the patient was given the option of open reduction and internal fixation (ORIF) of the fracture and elected to proceed. Unfortunately, given the loss of reduction, the patient’s pain was significant enough that she requested another closed reduction be performed until a definitive surgical reduction could be performed later that week. This request was reasonable, given her displacement and symptom improvement with the previously closed reduction performed in the ED. Pertinent medications were hydrocodone and acetaminophen; she was not actively taking any angiotensin-converting enzyme (ACE) inhibitors, statins, or diuretics.

**Figure 2:**
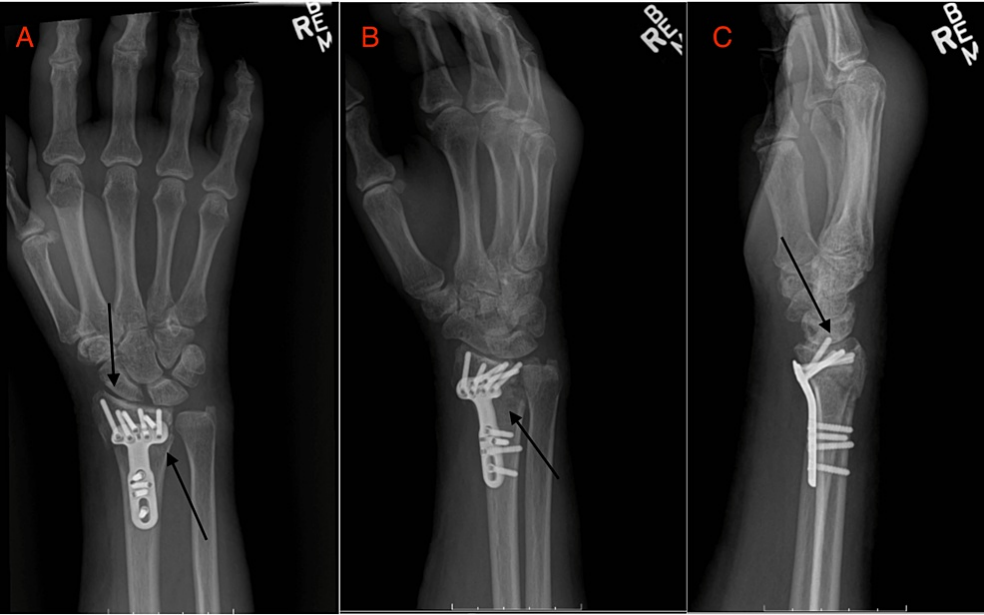
Posteroanterior (A), oblique (B), and lateral (C) radiographs of the right wrist taken postoperatively. A volar locking plate is in place, and anatomic parameters such as volar tilt, radial inclination, and radial height have been restored.

Verbal consent was obtained from the patient to attempt a closed reduction of the DRF at the bedside. The dorsal aspect of the wrist was prepped with an alcohol pad, and a hematoma block was administered into the fracture hematoma from a dorsal approach using a 21-gauge needle and 20 mL of 1% lidocaine without epinephrine. This was within the clinically accepted safe dose of 4.5 mg/kg. The injection was administered over the course of 30 seconds. Approximately one minute after the administration of lidocaine, the patient started to experience slurred speech and difficulty opening her eyes. She was able to articulate that she was seeing flashes of black and red. A Mini-Mental State Examination (MMSE) was performed, with the patient scoring 24 (normal is >25). The patient’s vitals, including heart rate and blood pressure, were normal (85 and 120/58, respectively). Due to concerns about systemic lidocaine administration, emergency medical transport was contacted. Within a couple of minutes of the onset of the patient’s symptoms, her speech improved, and she was able to open her eyes and follow commands. Her MMSE returned to normal, and emergency medical personnel were called off. The patient was then monitored in the clinic over the next hour, resulting in a complete resolution of her symptoms. It was recommended that the patient undergo further evaluation in the emergency department adjacent to the clinic, but the patient declined, given the rapid improvement in her symptoms. The closed reduction was not performed, given the events described, and the patient was taken to the operating room later that week for definitive treatment of her fracture (Figure 2).

## Discussion

Hematoma blocks are a simple and effective technique for providing anesthesia during the closed reduction of DRF in an acute setting [4]. The technique for administration involves the injection of a local anesthetic directly into the fracture hematoma. The dosage administered should be below the toxic weight-based dose of 4.5 mg/kg for lidocaine without epinephrine. This anesthetic is often the one of choice in this setting, as it has a relatively quick onset and a relatively short duration of effect. Additionally, it is of relatively low cost to both the patient and the treatment facility [5]. Our patient was a good candidate for a hematoma block but developed unexpected short-term neurologic complications.

A thorough review of the medical literature was conducted, and although several publications discuss intra-vascular local anesthetic complications and treatment, there is limited literature discussing neurological complications arising from hematoma blocks at the wrist [6,7]. A few case reports describing seizures or neurological symptoms associated with hematoma blocks at the wrist have been reported. One case report highlighted an 88-year-old female who experienced seizure-like symptoms following a hematoma block with 1% lidocaine and 0.25% bupivacaine at the wrist. The symptoms lasted five minutes before resolving. The patient did not experience any long-term neurological effects [6].

Lidocaine is a popular anesthetic due to its low cost and risk profile if given within an acceptable dose range. In the clinical setting, it can be used locally as an anesthetic, in the treatment of chronic pain syndromes, and systemically in the treatment of certain ventricular arrhythmias [8]. The mechanism of action of lidocaine is that it reversibly binds to voltage-gated sodium channels with a high affinity. The binding reduces both the production and propagation of action potentials in neurons. Lidocaine is also shown to be lipophilic and can quickly diffuse through the endothelial cells of the blood-brain barrier (BBB) when administered intravascularly [9-11]. However, the actual mechanism of action for BBB passage is subject to debate. There is literature to support lidocaine's inhibitory interaction with gamma-aminobutyric acid (GABA), which causes increased excitability leading to seizures [12]. In general, local toxicity can be treated through lipid emulsion [13-15]. However, most patients receiving DRF hematoma blocks with lidocaine do not experience neurological symptoms, making our patient's symptoms unusual.

We believe the symptoms experienced by the patient in this case report were secondary to the intravascular absorption of lidocaine through the cancellous channels of the radius with subsequent passage through the BBB. Given that the medication was administered dorsally at the level of the distal radius fracture, it is unlikely that it was administered directly intravascularly via the radial or ulnar arteries. It was postulated that the patient could have had an unusual anatomy, such as an anatomic variant of the radial artery, or an aberrant artery, such as a median artery; however, these variations were not visualized during the surgical procedure carried out later that week. As such, our current explanation of the patient’s symptoms is that the lidocaine was absorbed through the cancellous bone vascular channels and into the systemic venous circulation with subsequent passage through the BBB, leading to neurological complications. The relatively short duration of symptoms also supports this theory, though we cannot rule out a vasovagal response or other panic-induced disorders.

The outpatient orthopedic clinic is ill-equipped to manage the neurologic complications of intravascular local anesthetic absorption. In settings that lack lipids or other means to reverse the systemic effects of intravascular local anesthetics, the risks and benefits of performing hematoma blocks must be weighed. We no longer offer closed reduction as a temporary means of pain relief in patients ultimately requiring surgical fixation as definitive management of DRF. The lack of guidelines and literature discussing abnormal reactions to hematoma blocks is significant. This case report should raise awareness of this potential complication for all providers using this technique to treat displaced DRF. Going forward, the goal should be to create proper guidelines for patients who are candidates for this technique, discuss the risk of systemic intravascular absorption of the medication as a potential complication, and ensure that providers that use this technique have the availability of lipids nearby to treat this possible complication.

## Conclusions

Although commonly used to treat displaced distal radius fractures, hematoma blocks can have significant complications. Inadvertent intravascular administration of local anesthetic via venous channels in the cancellous bone is one potentially devastating complication that is not often discussed. Patients should be counseled about this potential complication when obtaining consent for the procedure. Emergency departments and other acute care facilities that use this technique to perform a closed reduction of fractures should be equipped with lipids to manage this potentially serious complication. This technique should be avoided in patients likely to require surgical intervention as a definitive treatment for their fracture.
